# Cyperenoic acid suppresses osteoclast differentiation and delays bone loss in a senile osteoporosis mouse model by inhibiting non-canonical NF-κB pathway

**DOI:** 10.1038/s41598-018-23912-3

**Published:** 2018-04-04

**Authors:** Supatta Chawalitpong, Ratchanaporn Chokchaisiri, Apichart Suksamrarn, Shigeru Katayama, Takakazu Mitani, Soichiro Nakamura, Ahmad AI Athamneh, Patcharee Ritprajak, Asada Leelahavanichkul, Ratchaneevan Aeimlapa, Narattaphol Charoenphandhu, Tanapat Palaga

**Affiliations:** 1 Graduate Program in Biotechnology, Faculty of Science, Chulalongkorn University, Phayathai Road, Pathumwan, Bangkok, 10330 Thailand; 2 Department of Chemistry, School of Science, University of Phayao, Muang, Phayao, 56000 Thailand; 3 Department of Chemistry and Center of Excellence for Innovation in Chemistry, Faculty of Science, Ramkhamhaeng University, Ramkhamhaeng Road, BangKapi, Bangkok, 10240 Thailand; 4 Department of Bioscience and Biotechnology, Shinshu University, 8304 Minamiminowa, Kamiina, Nagano, Japan; 5 Institute for Biomedical Sciences, Interdisciplinary Cluster for Cutting Edge Research, Shinshu University, 8304 Minamiminowa, Kamiina, Nagano, Japan; 6 Department of Microbiology and Immunology and Research Unit of Oral Microbiology, Faculty of Dentistry, Chulalongkorn University, Bangkok, 10330 Thailand; 7 Department of Microbiology, Faculty of Medicine, Chulalongkorn University, Phayathai Road, Pathumwan, Bangkok, 10330 Thailand; 8 Department of Physiology, Faculty of Science, Mahidol University, Rama 6 Road, Bangkok, 10400 Thailand; 9 Center of Calcium and Bone Research (COCAB), Faculty of Science, Mahidol University, Bangkok, 10400 Thailand; 10 Institute of Molecular Biosciences, Mahidol University, Nakhon Pathom, Thailand; 11 Department of Microbiology, Faculty of Science, Chulalongkorn University, Bangkok, Thailand; 12 Center of Excellence in Immunology and Immune-mediated Diseases, Chulalongkorn University, Phayathai Road, Pathumwan, Bangkok, 10330 Thailand

## Abstract

Cyperenoic acid is a terpenoid isolated from the root of a medicinal plant *Croton crassifolius* with a wide range of biological activities. In this study, the effects of cyperenoic acid on osteoclast differentiation were investigated both *in vitro* and *in vivo* using receptor activator of nuclear factor-κB ligand (RANKL)-induced bone marrow-derived osteoclasts and senescence-accelerated mouse prone 6 (SAMP6). Cyperenoic acid significantly suppressed RANKL-induced osteoclast differentiation at the concentrations with no apparent cytotoxicity. The half maximum inhibitory concentration (IC_50_) for osteoclast differentiation was 36.69 μM ± 1.02. Cyperenoic acid treatment evidently reduced the expression of two key transcription factors in osteoclast differentiation, NFATc1 and c-Fos. Detailed signaling analysis revealed that cyperenoic acid did not affect MAPK pathways and canonical NF-κB pathway but impaired activation of p100/p52 in the non-canonical NF-κB pathway upon RANKL stimulation. Moreover, the expression of osteoclast-related genes, *nfatc1*, *ctsk*, *irf8*, *acp5* and *cfos* were disrupted by cyperenoic acid treatment. The bone resorption activity by cyperenoic acid-treated osteoclasts were impaired. In a senile osteoporosis mouse model SAMP6, mice fed on diet supplemented with cyperenoic acid showed delay in bone loss, compared to the control. Taken together, plant-derived cyperenoic acid shows great potential as therapeutic agent for osteoporosis.

## Introduction

Osteoporosis is one of the major health concerns for aging communities. There are two types of osteoporosis which are postmenopausal osteoporosis occurred in woman after menopause and senile osteoporosis occurred in both men and women often over 70 years of age^1^. The balance between functions of osteoblasts and osteoclasts is essential for maintaining bone homeostasis. When this balance is disrupted by various conditions such as menopause, a progressive decrease in bone mass manifests and this condition leads to an increased susceptibility to bone fractures^2,3^.

Macrophage-colony stimulating factor (M-CSF) and receptor activator of nuclear factor-B ligand (RANKL) provide two necessary signals for osteoclast differentiation^4^. The signals generated by these two major cytokines depend on and converge at mitogen activated protein kinase (MAPK) pathway (p38, SAPK/JNK and P44/42)^5^, and NF-κB pathways. The NF-κB pathway bifurcate via a canonical pathway mediated through inhibitor of IκB kinase (IKK) and p65 and RelA/p50^6^ and non-canonical pathway involved IKKα, NF-κB inducing kinase (NIK) and RelB/p52^7^. The costimulatory signaling via PLCγ-Ca^2+^ is also crucial for osteoclast differentiation program^8^. These downstream early intracellular signaling culminates in activation of key transcription factors in osteoclastogenesis, including c-Fos, AP-1 and NFATc1^2^. Among these transcription factors, NFATc1 plays essential roles in osteoclast differentiation^9^.

The non-canonical NF-κB pathway is one arm of the NF-κB signaling pathway which plays an important role in osteoclastogenesis. Various cytokines signal through this pathway, including RANKL, LTβ and CD40L^10^. Upon receptor/ligand interaction, the signaling is initiated by recruitment of adapter protein TNF receptor-associated factor (TRAF) 2 and TRAF3 to the receptors. TRAF3 is a negative regulator that suppresses the activation of NIK by targeting it for degradation via ubiquitination^10^. In the case of RANK/RANKL stimulation, TRAF3 is ubiquitinated and degraded by cellular inhibitor of apoptosis (cIAP) 1 and 2 leading to accumulation of NIK, which in turn, activates IKKα^10^. Activated IKKα triggers phosphorylation and partial degradation of p100, resulting in the mature form of p100 (p52). The signaling controls the expression of various genes by p52 which can form a dimer with another NF-κB subunit, RelB, and translocate to nuclei and activate the gene expression^11^. Targeted deletion of NF-κB p50/p52 in mice recapitulates the phenotype of RANK/RANKL knock out animals, indicating crucial roles these pathways play in RANKL-mediated osteoclast differentiation^12,13^.

Senescence Accelerated Mouse Prone 6 (SAMP6) developed by Takada *et al*. (1981)^14^ from AKR/J strains is a useful mouse model of senile osteoporosis. They are characterized by a low peak in bone mass early on after the age of 16 weeks^15^. The decreasing in bone formation and the increasing in bone resorption in SAMP6 is postulated to be caused by deficiency in osteoblast progenitor cells^14^ and defects in maturation of osteoclast^16^. In a study by Shimizu *et al*.^17^, they reported an abnormality in SAMP6 mice of a group of genes located at *Pbd2* locus, on chromosome 13^17^. The appearance of SAMP6 mice is similar to that found in aging human^18^. Therefore, SAMP6 is proposed to be one of senile osteoporosis animal models^19,20^.

Effective treatment of osteoporosis is urgently needed as the world population in various parts of the world live longer. Plants derived compounds are rich sources for identification of lead compounds for drug development. *Croton crassifolius* Geisel (Euphorbiaceae) is a medicinal plant distributed mainly in Southern East Asia. The main phytochemical compound in *C*. *crassifolius* consists of diterpenoids^21^ and sesquiterpenes. Cyperenoic acid belongs to the major sesquiterpenes which are isolated from the root of *C*. *crassifolius*^22^. Huang *et al*.^23^ reported that cyperenoic acid shows activity against angiogenesis in the zebrafish embryo model^23^. In addition, cyperenoic acid suppresses the releasing of vascular endothelial growth factor (VEGF) in MCF-7 and HepG2 cancer cell lines^24^. However, until now there is no report on the effect of cyperenoic acid on osteoclastogenesis.

In this study, we reported that cyperenoic acid has strong anti-osteoclastogenic activity in an *in vitro* model of RANKL-induced bone marrow (BM)-derived osteoclast differentiation. Detailed molecular mechanism identified the non-canonical NF-κB pathway as the target of this naturally occurring terpenoid. Furthermore, its effect on bone loss in SAMP6 mice was investigated.

## Materials and Methods

### Bioactive compound and antibodies

**Figure 1:**
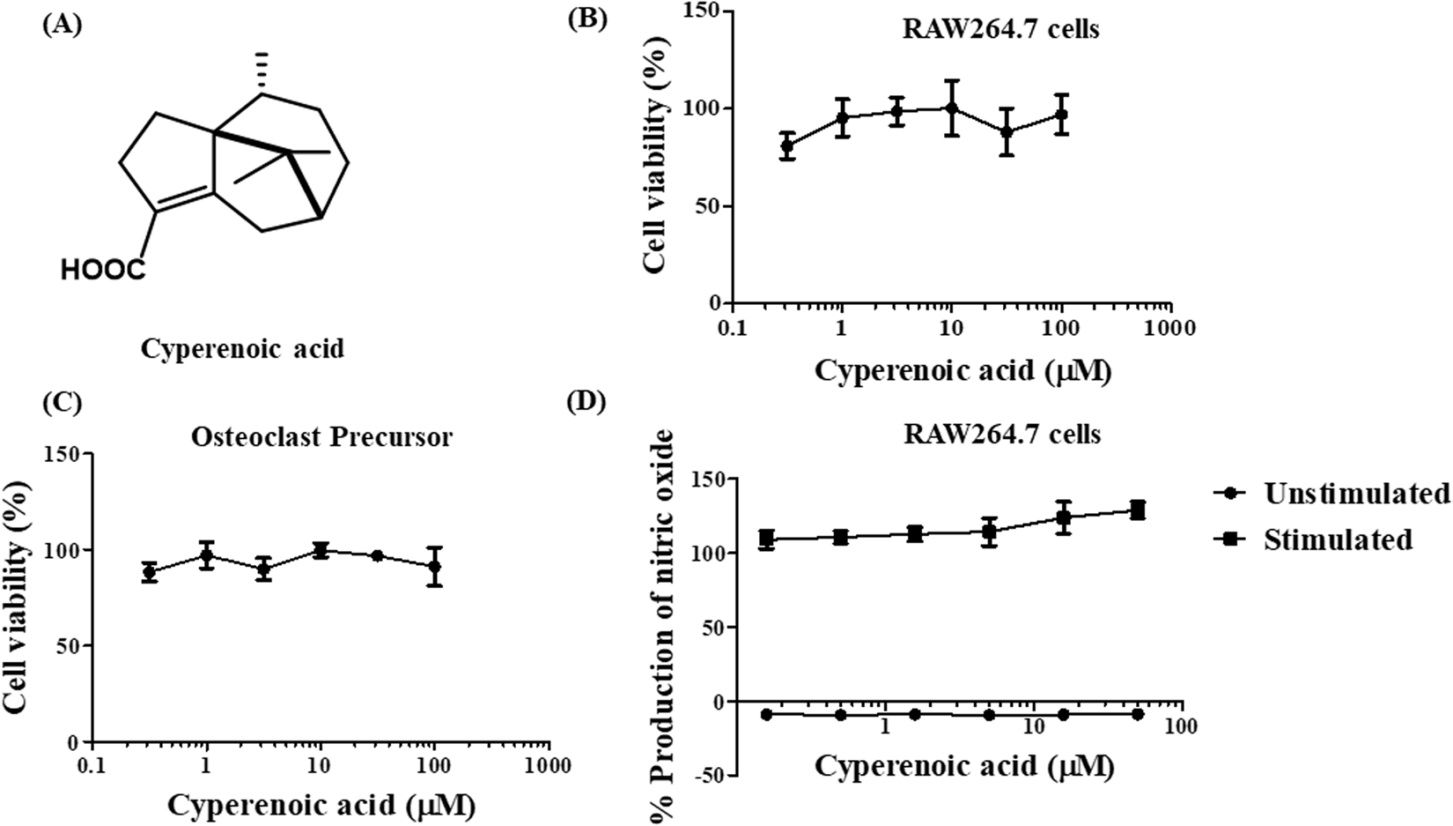
Structure and effect of cyperenoic acid on cell viability and inflammatory response in RAW264.7 macrophage like cell line. (**A**) The chemical structure of cyperenoic acid isolated from *Croton crassifolius* is shown. (**B**) RAW264.7 cells or (**C**) BMs-derived osteoclast precursors were treated with various concentrations of cyperenoic acid for 24 h and cell viability was determined by MTT assay. The IC_50_ for cell viability is ≥100 µM. (**D**) RAW264.7 cells were stimulated with or without LPS (100 ng/ml) and IFN-γ (10 ng/ml) in the presence of various concentrations of cyperenoic acid or vehicle control DMSO. The amount of nitric oxide produced in the culture supernatant was measured. The data are representative of three independent experiments and presented as the mean ± S.D.

Cyperenoic acid (Fig. 1A) was isolated from the roots of *C*. *crassifolius*. The roots of this plant species were collected from Nacharoen, Dech Udom district, Ubon Ratchathani Province, Thailand, in December 2009. A voucher specimen (Apichart Suksamrarn, No. 062) is deposited at the Faculty of Science, Ramkhamhaeng University (Thailand). Antibodies against p38, phospho-p38, SAPK/JNK, phospho-SAPK/JNK, ERK (p44/42), phospho-ERK (pERK), NF-κB p65, phospho-p65, IKB-α, NF-κB p100/52, phospho-p100, TRAF3, RelB, c-Fos, NFATc1 were purchased from Cell Signaling Technology (MA, USA). Antibody against β-actin was from Merck Millipore (MA, USA).

### Cell culture

A macrophage-like cell line, RAW264.7 (ATCC TIB-71) and pre-osteoblast cell line, MC3T3-E1 (RBRC-RCB1126) were maintained in DMEM medium (Thermo Fisher Scientific, MA, USA) and α-MEM medium (GIBCO, no A1049001 UK), respectively. The media were supplemented with 10% Fetal Bovine Serum (GIBCO-Invitrogen, USA), 1% HEPES free acid, 1% sodium pyruvate and 1% penicillin-streptomycin (Thermo Fisher Scientific, UK). Cells were maintained at 37 °C in 5% CO_2_ incubator (Thermo Electron Corporation, USA). BMs were isolated from humerus, femur and tibia of 6–8 weeks old BALB/c female mice (National Laboratory Animal Center, Mahidol University). BMs were cultured with M-CSF (25 ng/ml; Immunotools, Germany) for 48 h to obtain osteoclast precursors. For osteoclast differentiation, osteoclast precursors were further cultured with M-CSF and RANKL (100 ng/ml; Biolegend, CA, USA) for indicated times. All procedures involving laboratory animals were approved by the Institutional Animal Care and Use Committee of Chulalongkorn University.

### Cell viability and anti-inflammation assay

Cell lines, RAW264.7, MC3T3-E1 (1 × 10^4^ cells/well in 96 well plates) or BMs (1 × 10^5^ cells/well in 96 well plates) were treated with cyperenoic acid or vehicle control, at indicated concentrations for 24 h. MTT solution (Alfa Aesar, UK) was used to determine the cell viability. The relative cell viability (%) was expressed as percentages relative to the untreated control cells. The IC_50_ was calculated using a GraphPad Prism 5.03 (GraphPad Software, USA). For anti-inflammation activity, RAW264.7 cells were treated with cyperenoic acid and stimulated with or without lipopolysaccharide (LPS) (100 ng/ml, Sigma Aldrich, MO, USA) and recombinant murine interferon-gamma (IFN-γ) (10 ng/ml, BioLegend, CA, USA) for 24 h. The amount of nitric oxide produced were measured by Griess reaction. The relative nitric oxide productivity (%) was expressed as a percentage relative to the untreated control and compared with nitrite standard.

### Osteoclast differentiation by tartrate-resistant acid phosphatase (TRAP) staining assay

Osteoclast differentiation from murine bone marrow cells were performed as described elsewhere with some modification^25^. Briefly, BMs-derived osteoclast precursors (3 × 10^6^ cells/well in 60 mm culture tissue culture dish) were seeded and incubated with M-CSF (25 ng/ml) for 48 h to induce differentiation of osteoclast precursors. The precursors of osteoclasts were harvested by cold PBS and seeded at 2.5 × 10^5^ cells/well in 96-well plates. Cells were pre-treated with cyperenoic acid or vehicle control DMSO for 30 min and stimulated with RANKL (100 ng/ml) and M-CSF (25 ng/ml) for 5 days. After the incubation, cells were washed and fixed with 10% formaldehyde. Cells were stained with a TRAP-staining solution (50 mM acetate buffer, contained with 50 mM sodium tartrate, 0.1 mg/ml naphthol AS-MX phosphate and 0.6 mg/ml Fast red violet LB salt). TRAP+ multinucleated cells (MNCs) with three or more nuclei per cell were counted as mature osteoclast under a light microscope (Olympus, Tokyo, Japan).

### Expression of osteoclast related genes by qPCR

Total RNA was isolated by using Trizol reagent (Thermo Fisher Scientific, MA, USA) or RNAiso reagent (Takara-Bio, Japan) according to the manufacturer’s instruction. Isolated RNA was measured using Quan iT assays (Thermo Fisher Scientific, MA, USA). Primers for osteoclastogenic genes used in this study is listed in the Supplementary Table 1. A SYBR^®^ Green (Bio-Rad, USA) or Kapa SYBR (Kapa Biosystems, USA) was used for qPCR. Relative expression of osteoclast markers was determined and normalized, using the expression levels of *β-actin* or *gapdh* and calculated by 2^−ΔΔCt^ method^26^.

### Immunoblot

Cellular proteins were extracted using RIPA buffer and the amounts were measured by using Pierce BCA Protein Assay Kit (Thermo Fisher Scientific, MA, USA). Proteins were separated with 8% or 12% SDS-PAGE and transferred onto PVDF membranes (Merck Millipore, MA, USA). The membranes were blocked with 3% non-fat-milk in PBS-T or 5% BSA in TBS-T and then probed with indicated antibodies. The signals were visualized by chemiluminescence with high performance chemiluminescence X-ray film.

### Bone resorption assay

Osteoclast differentiation from murine bone marrow cells were performed as described elsewhere with some modification^25^. Briefly, osteoclast precursors were seeded in 96-well plates containing dentine or bone slices (IDS^®^ Immunodiagnostic Systems, UK) (2.5 × 10^5^ cells/well), and stimulated with RANKL (100 ng/ml) and M-CSF (25 ng/ml) for 14 days. Cyperenoic acid (100 μM) or vehicle control DMSO was added to the culture at indicated times. At the end of the culture period, all cells were removed by sonication for 15 seconds in concentrated ammonium hydroxide and washed with double distilled water. The dentine or bone slices were stained for 10 min by Toluidine Blue (Sigma Aldrich, MO, USA), followed by extensive washing. The resorption areas were examined by a light microscope and photographed. The percentage of the resorbed areas were determined using Image-J software, area measurement, at 100-fold magnification.

### Osteoblast differentiation by alkaline phosphatase activity (ALP) assay

Osteoblast differentiation using MC3T3-E1 cell line were performed as described elsewhere^27^. Briefly, MC3T3-E1 cell line (2.5 × 10^4^ cells/well in 12-well plates) was induced to differentiate to osteoblasts by ascorbic acid (200 μM, Sigma Aldrich, MO, USA) or ascorbic acid in combination with β-glycerophosphate (8 mM, Sigma Aldrich, MO, USA) for 10 days. During this period, media were changed every 3 days. For ALP assay, cells were washed and harvested with 1xPBS and 0.1% triton x-100 for 10 min, respectively. The protein content was measured by using the BCA assay. Normalized protein lysates were incubated with 0.1 M sodium bicarbonate-carbonate buffer (pH 10) containing with 2 mM MgSO_4_ and 6 mM p-nitrophenol phosphate for 30 min at 37 °C. The reaction was stopped by using 1.5 M NaOH (1:1 v/v) and absorbance was measured at 405 nm by microplate reader (Thermo Fisher Scientific, MA, USA).

### A senile osteoporosis mouse model SAMP6

Female Senescence Accelerated Mouse Prone 6 (SAMP6) (7 weeks old) weight between 35–40 g were purchased from Japan SLC, Inc. (Shizuoka, Japan). They were allowed to acclimatize for 1 week before the start of the experiment. All mice were randomly separated into two groups of control and cyperenoic acid treated group (6 mice per group). All experiments involved SAMP6 mice were conducted in accordance with the institutional guideline established by Shinshu University for laboratory animals.

The control group was fed with a standard rodent diet AIN-93M mixed with absolute ethanol (Oriental Yeast, Tokyo, Japan) and the cyperenoic acid treated group was fed with a standard diet mixed with 0.01% (w/w) cyperenoic acid powder. Mice were allowed free access to the diet and water. Food consumption and body weight were measured once a week. After 19 weeks, SAMP6 mice long bones (humerus, femur and tibia) were collected for BMs to be used to detect gene expression and for bone histomorphometry. Kidneys, livers and spleens were collected for pathohistological examination.

### Bone histomorphometry

Bone histomorphometry was performed according to the modified methods of Orris and Arnett^28^ and Vidal *et al*.^29^. Mice femur bones were fixed with 4.5% formaldehyde (pH 7.2) for 2 days, decalcified with 14% EDTA (pH 7.2) for 14 days and dehydrated with 70%, 80%, 96% and 100% ethanol, respectively. The processed bones were embedded in paraffin and the embedded bones were sectioned by microtome to a 5-µm thickness. The bone tissue sections were stained with hematoxylin, fast green CFC and safranin O and examined by a light microscope (model BX51TRF; Olympus, Tokyo, Japan). The analyzed areas covered the trabecular region (i.e., secondary spongiosa) of distal femur at 500 µm distal to the growth plate (orange color). The static histomorphometric parameters obtained from each section were bone volume/tissue volume (also known as bone volume fraction; BV/TV; %), trabecular thickness (Tb.Th; µm), trabecular separation (Tb.Sp; µm) and trabecular number (Tb.N; mm^−1^). These histomorphometric parameters were analyzed by the computer-assisted OsteoMeasure system with software version 7 (OsteoMetrics, Atlanta, USA), according to the standard of the ASBMR Histomorphometry Nomenclature Committee.

### Statistical analyses

All experiments were repeated independently three times unless otherwise specified. The results were expressed as the mean ± standard deviation (SD). The statistical significance of difference between an experimental group and its corresponding control were evaluated by one-way ANOVA, two-way ANOVA or Student’s T-test (GraphPad Prism 5.03). All comparisons were made as specified, and *p* < 0.05 was considered statistical significance.

## Results

### Cytotoxicity and anti-inflammatory activity of cyperenoic acid

To determine the toxicity of cyperenoic acid on macrophage-like cell line RAW264.7 or BMs-derived osteoclast precursors, cells were treated with cyperenoic acid at various concentrations (0–100 μM) and the cell viability was measured. As shown in Fig. 1B,C, cyperenoic acid had no toxicity at the concentration as high as 100 μM in RAW264.7 or osteoclast precursors. Therefore, this concentration was used in further experiments. To examine the effect of cyperenoic acid on inflammatory response, the effect on nitric oxide production in RAW264.7 cell line stimulated with LPS/IFN-γ for 24 h was examined. As shown in Fig. 1D, this compound did not show any effect on nitric oxide production in LPS/IFN-γ-stimulated macrophages.

### Anti-osteoclast differentiation activity of cyperenoic acid

**Figure 2:**
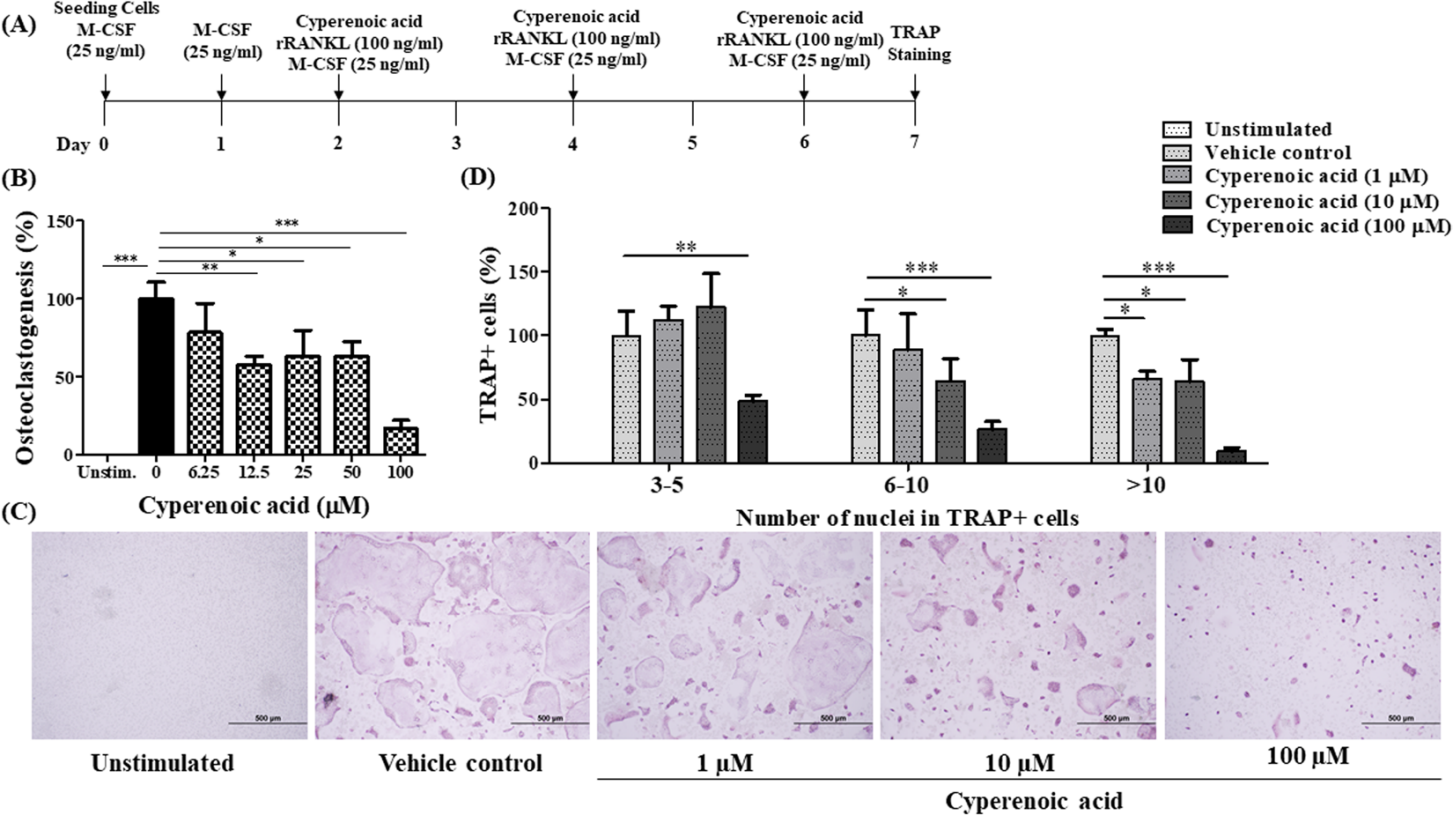
Effect of cyperenoic acid on RANKL-induced osteoclast differentiation. (**A**) A procedure used to evaluate the effect of cyperenoic acid on RANKL-induced osteoclast differentiation is shown. BMs were cultured with rRANKL (100 ng/ml) and M-CSF (25 ng/ml) in the presence of cyperenoic acid or vehicle control DMSO. Cells were stained for TRAP activity and TRAP+ MNCs were counted. (**B**) BMs were treated with various concentrations of cyperenoic acid (0–100 µM). The IC_50_ of appearance of TRAP+ MNCs for cyperenoic acid was 36.69 µM ± 1.02. (**C**) TRAP staining patterns of osteoclasts are shown. (**D**) BMs were treated as described in (**A**) in the presence of cyperenoic acid (0, 1, 10 and 100 µM). The TRAP+ MNCs were counted based on the number of nuclei in each cell and the percentage of cells with the indicate range of nuclei per cells were calculated. The data are representative of three independent experiments and presented as the mean ± S.D. ***p < 0.001, **p < 0.01 and *p < 0.05 indicated statistical significance.

A procedure to test the activity of cyperenoic acid against osteoclast differentiation was shown in Fig. 2A. To determine the dose dependent activity, various concentrations of cyperenoic acid were used. As shown in Fig. 2B, the percentages of differentiated osteoclasts as judged by TRAP+ MNCs, decreased upon cyperenoic acid treatment in a dose dependent manner. The IC_50_ for osteoclastogenesis was 36.69 µM ± 1.02. The highest concentration (100 μM) used to determine an IC_50_ completely suppressed RANKL-induced osteoclast differentiation (Fig. 2C). To test whether cyperenoic acid treatment affects cell-cell fusion, a hall mark step of osteoclast differentiation, BMs were treated with cyperenoic acid as described above and the TRAP+ MNCs were counted and divided into cells with 3–5 nuclei, 6–10 nuclei or more than 10 nuclei. As shown in Fig. 2C, 2D, cyperenoic acid treatment significantly reduced the percentages of cells with 3 or more nuclei per cell in a dose dependent manner. Therefore, cyperenoic acid showed strong inhibitory activity against RANKL-induced osteoclast differentiation in murine BMs.

### Effect of cyperenoic acid on expression of osteoclast-related genes in osteoclast precursors

**Figure 3:**
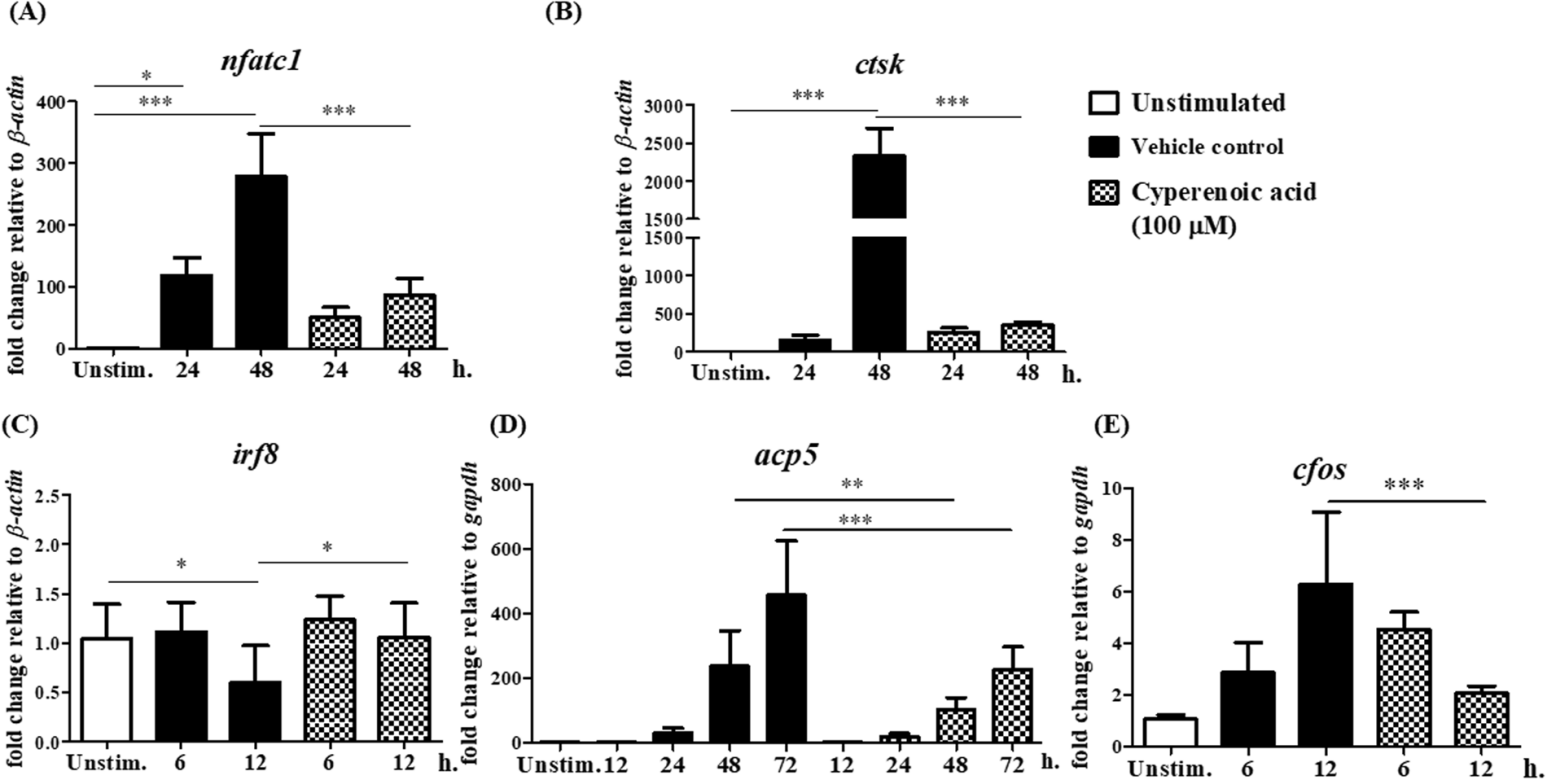
Effect of cyperenoic acid on expression of osteoclast-related genes. BMs were pre-treated with M-CSF with DMSO and left unstimulated (open bars), M-CSF with DMSO for 30 min, followed by RANKL stimulation (closed bars) or M-CSF with cyperenoic acid (100 µM) for 30 min, followed by RANKL stimulation (hatched bars) for indicated times. Total RNA was extracted and subjected to RT-qPCR. Osteoclast-related genes were *nfatc1* (**A**), *ctsk* (**B**), *irf8* (**C**), *acp5* (**D**) and *cfos* (**E**). The data are representative of three independent experiments and presented as the mean ± S.D. ***p < 0.001, **p < 0.01 and *p < 0.05 indicated statistical significance.

As the first step to investigate how cyperenoic acid interferes with RANKL-induced osteoclast differentiation, its effect on expression of osteoclast-related genes (*nfatc1*, *ctsk*, *irf8*, *acp5* and *cfos*) were determined. BMs were pre-treated with cyperenoic acid (100 μM) or vehicle control DMSO for 30 min and then stimulated with RANKL for various durations as indicated in Fig. 3. As shown in Fig. 3A, treatment of cyperenoic acid completely abrogated the expression of *nfatc1* both at 24 and 48 hr. Because a gene encoding cathepsin K, *ctsk*, is under the regulation of NFATc1, the mRNA level of *ctsk* was examined. As shown in Fig. 3B and Supplementary Figure 1, treatment with cyperenoic acid clearly reduced the level of *ctsk* mRNA at 48 h to almost the base line level. IRF8 is an inhibitory factor functioning to inhibit osteoclast differentiation and its level must be reduced to allow osteoclast differentiation^30^. As expected, cyperenoic acid treatment delayed the downregulation of *irf8* at 12 h (Fig. 3C). The levels of additional two genes, i.e. *acp5* and *cfos*, that are involved in osteoclast function and differentiation were examined. The mRNA levels for both genes were significantly reduced in the presence of cyperenoic acid (Fig. 3D, E). Taken together, the expression of genes involved in an early stage of osteoclast differentiation was compromised by cyperenoic acid treatment.

### Effect of cyperenoic acid on signaling pathways downstream of RANK/RANKL

**Figure 4:**
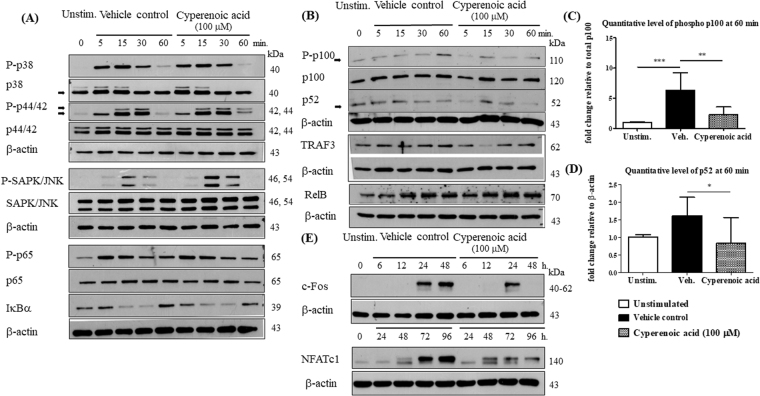
Effect of cyperenoic acid on activation of MAPK, canonical or non-canonical NF-κB pathways and expression of NFATc1 and c-Fos. M-CSF treated BMs were pre-treated with cyperenoic acid (100 μM) or vehicle control DMSO for 30 min and RANKL (100 ng/ml) was used to stimulate cells at the indicated times. Whole cell lysates were analyzed for phosphorylation forms of MAPK and canonical NF-κB (**A**) or non-canonical NF-κB (**B**) by Western blot. (**C**–**D**) The quantitative band density of phosphorylated p100 and total p52 were normalized by total p100 or β-actin, respectively. (**E**) The level of c-Fos and NFATc1 were analyzed by Western blot. β-actin was used as a loading control. Data were representative of three independent experiments.

To examine the effect of cyperenoic acid on the signaling cascades downstream of RANK upon RANKL engagement, the phosphorylation of signaling molecules in the MAPK and canonical NF-κB pathways were detected. BMMs were pre-treated with cyperenoic acid (100 μM) or vehicle control DMSO for 30 min, and stimulated with RANKL at indicated time points. As shown in Fig. 4A, phosphorylation of signaling molecules in the MAPK and canonical NF-κB pathways appeared unaltered in the presence of cyperenoic acid after RANKL stimulation. In contrast, when the activation of non-canonical NF-κB pathway was investigated, treatment with cyperenoic acid clearly suppressed the phosphorylation of p100 and the appearance of p52 at 5 and 60 min after RANKL stimulation (Fig. 4B, Supplementary Figure 2C). Quantitative analysis of band densities of phosphorylated p100 or p52 at 60 min confirmed that cyperenoic acid treatment reduced phosphorylation of p100 and the level of p52 (Fig. 4C,D). In addition, the level of TRAF3 was transiently reduced at 15 min in the presence of cyperenoic acid. In contrast, the effect of cyperenoic acid on RelB was only minimum. Taken together, cyperenoic acid affects mainly the activation of non-canonical NF-κB pathway but does not interfere with MAPK or canonical NF-κB pathway.

To further confirm the effect of cyperenoic acid on the expression of transcription factors regulated by these early downstream pathways, the protein levels of c-Fos and NFATc1 were detected by immunoblot. As shown in Fig. 4E and Supplementary Figure 2B, treatment with cyperenoic acid completely suppressed c-Fos expression at 48 h and impaired the NFATc1 expression at 72 and 96 h after RANKL stimulation. These data are consistent with the levels of transcripts of both genes observed in Fig. 3A and E.

### Effect of cyperenoic acid treatment on bone resorption by osteoclasts

**Figure 5:**
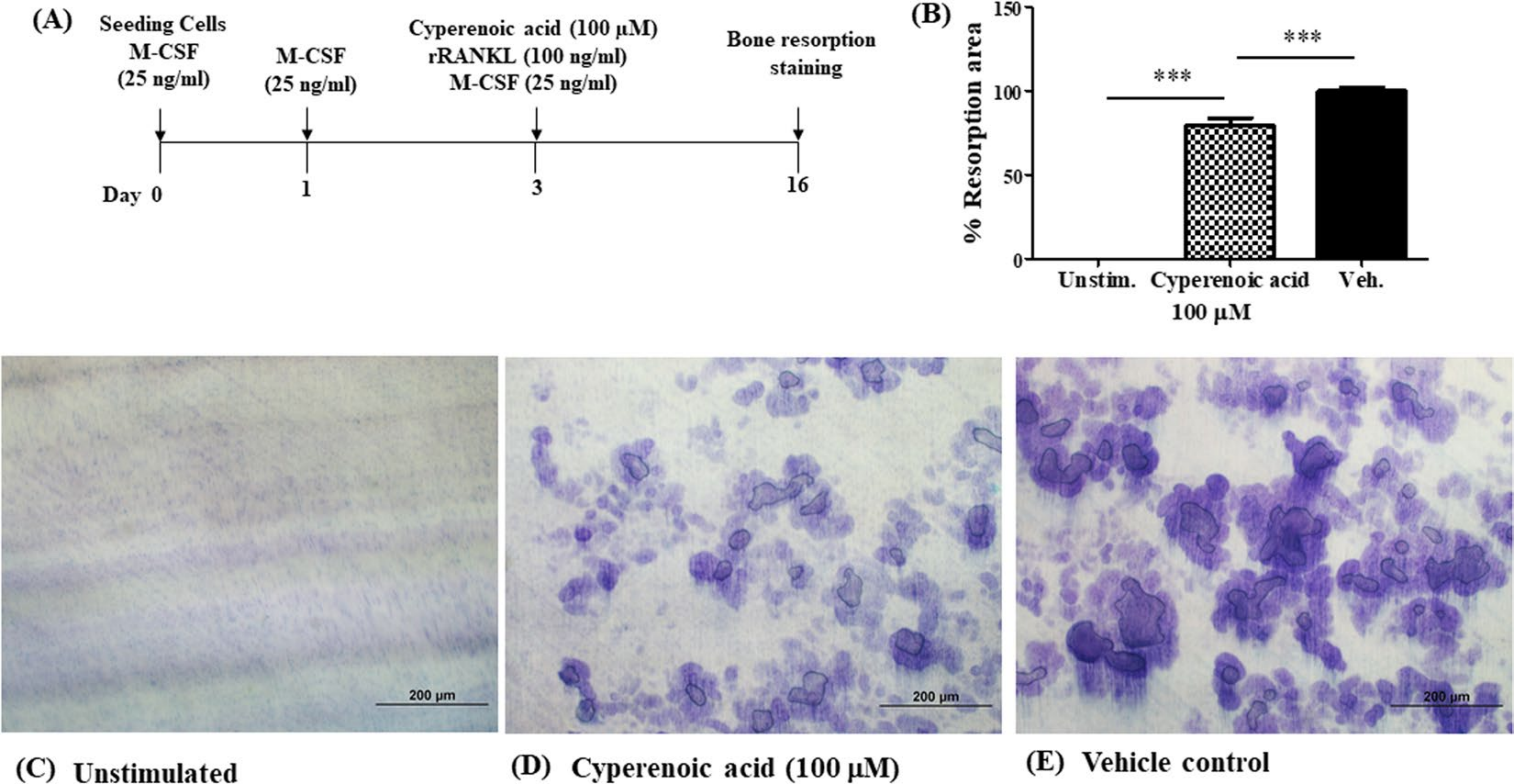
Effect of cyperenoic acid on bone resorption by precursor osteoclast. (**A**) A procedure used to evaluate the effect of cyperenoic acid on RANKL-induced osteoclast differentiation on dentin slices is shown. The resorption areas were stained with toluidine blue and observed by light microscope. (**B**) The resorption areas were measured and quantitated as a percentage of total resorbed area using Image-J software at 100-fold magnification of at least three unrelated fields. (**C**–**E**) The staining of bone resorption areas on dentin slices were shown. The data presented as the mean ± S.D. ***p < 0.001 indicated statistical significance.

To investigate whether cyperenoic acid treatment interrupts osteoclast function, dentine or bone slices were subjected to a bone resorption assay by osteoclasts differentiated in the presence or absence of cyperenoic acid for 14 days. First, cyperenoic acid was added from the start of osteoclast differentiation as schematically shown in Fig. 5A. As shown in Fig. 5B–E, cyperenoic acid treatment clearly reduced the area of osteoclast-mediated bone resorption, consistent with its negative effect on osteoclast differentiation. The areas of bone resorption were partially, but significantly, reduced when osteoclasts differentiated in the presence of cyperenoic acid were used for this assay.

**Figure 6:**
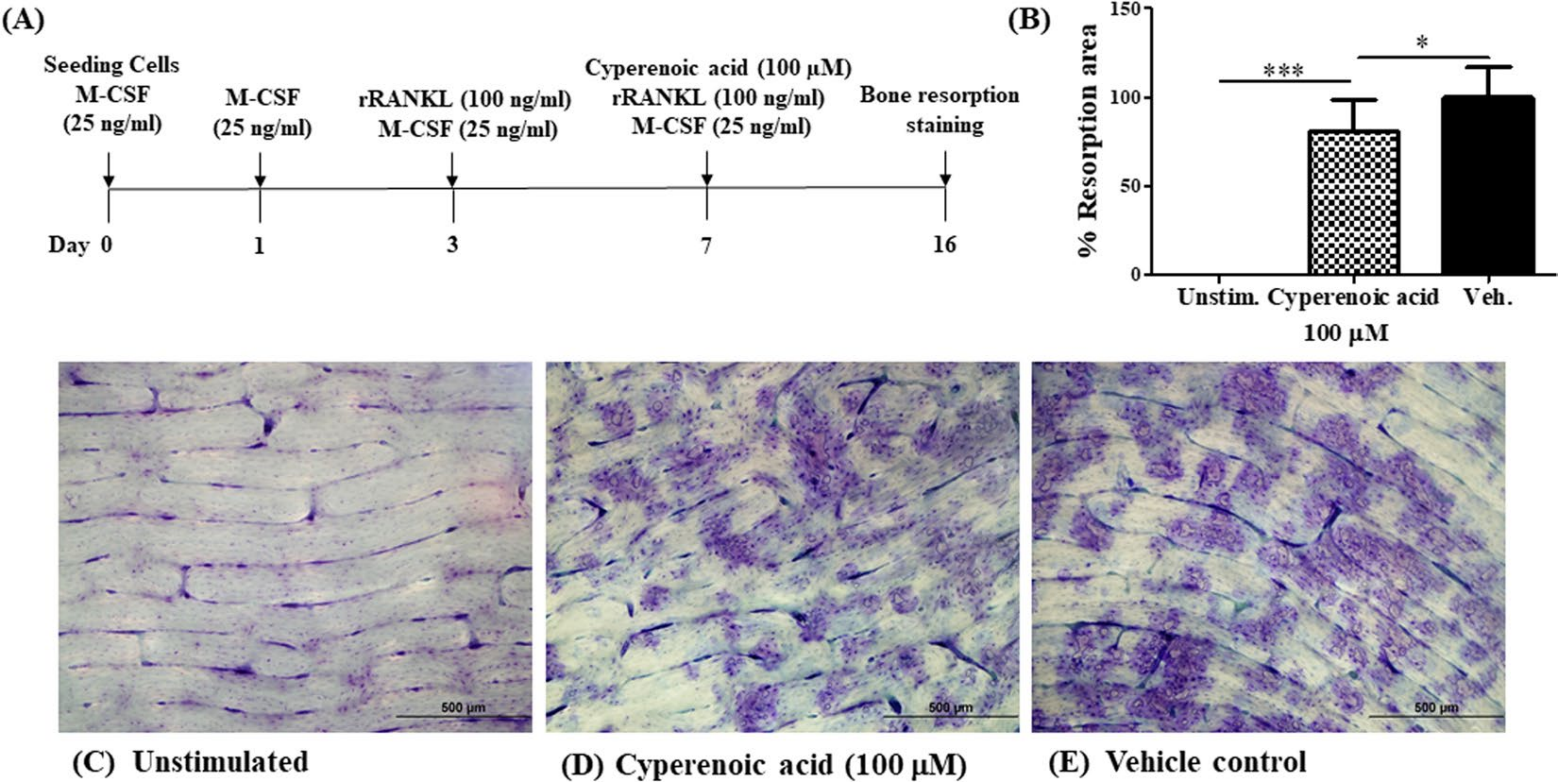
Effect of cyperenoic acid on bone resorption by mature osteoclast. (**A**) A procedure used to evaluate the effect of cyperenoic acid on bone resorption by RANKL-induced osteoclasts on bone slices is shown. (**B**) The resorption areas were stained with toluidine blue and the resorption areas were measured and quantitated as a percentage of total resorbed area using Image-J software at 100-fold magnification of at least three unrelated fields. (**C**–**E**) The staining of bone resorption areas on bone slices were shown. The data presented as the mean ± S.D. ***p < 0.001 and *p < 0.05 indicated statistical significance.

To evaluate whether cyperenoic acid treatment after osteoclast maturation interferes with the bone resorption functions of osteoclasts, cyperenoic acid was added to the culture at day 7 after initiation of osteoclast differentiation (Fig. 6A). The areas of bone resorption were measured as described above. As shown in Fig. 6B–E, cyperenoic acid treatment even when added after the completion of osteoclast differentiation, also impinged upon bone resorption activity of osteoclasts. Taken together, this evidence strongly indicated anti-bone resorption activities of cyperenoic acid.

### Effect of cyperenoic acid on osteoblast differentiation

Some compounds with anti-osteoclastogenesis also possess pro-osteoblastogenic activity. To evaluate this possibility, we determined the cytotoxicity and the effect of cyperenoic acid on osteoblast differentiation induced by ascorbic acid or ascorbic acid in combination with β-glycerophosphate in the pre-osteoblast cell line MC3T3-E1. The cytotoxicity of cyperenoic acid on the MC3T3-E1 cell was first evaluated with cyperenoic acid of various concentrations (0–100 μM). The result revealed no apparent toxicity of cyperenoic acid in this cell line within the range of tested concentrations (Supplementary Figure 3A). Furthermore, cyperenoic acid had no effect on ALP activity induced by ascorbic acid or ascorbic acid in combination with β-glycerophosphate in osteoblasts as shown in Supplementary Figure 3B and C. Thus, cyperenoic acid appears to affect only the differentiation and function of osteoclasts, but not differentiation of osteoblasts.

### Effect of cyperenoic acid on bone loss in SAMP6 mice of senescence-induced osteoporosis

SAMP6 mice were continually fed with normal diet or cyperenoic acid-supplemented diet for 19 weeks. The body weight and the diet consumption were measured once a week. As shown in Supplementary Figure 4A and B, there were no differences in the body weight and the diet consumption between both groups of mice. Furthermore, livers and kidneys were subjected to histopathological examination at the end of the experiment and no obvious toxicity to these tissues were found (Supplementary Figure 4C–F).

**Figure 7:**
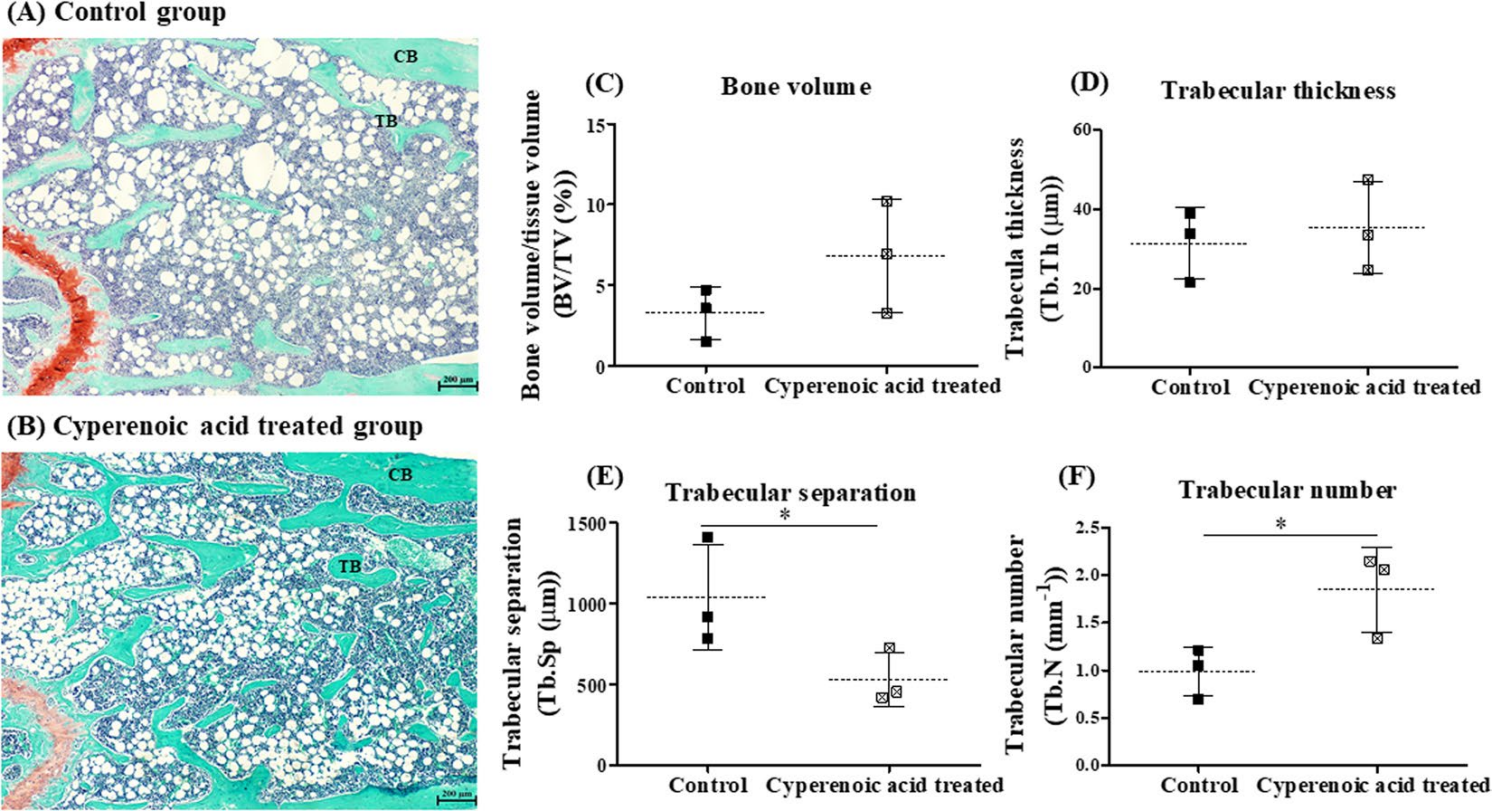
Effect of cyperenoic acid on bone histomorphometry at distal femur in SAMP6 mice. SAMP6 mice were fed with the standard diet food AIN-93M (**A**) or the standard diet food supplemented with 0.01% cyperenoic acid (**B**) for 19 weeks. Representative microscopic image of femoral bone at distal end stained with safranin O (orange), fast green (green) and hematoxylin (blue) are shown. Bone histomorphometric parameters of bone volume normalized by tissue volume (BV/TV) (**C**), trabecular thickness (Tb.Th) (**D**), trabecular separation (Tb.Sp) (**E**) and trabecular number (Tb.N) (F) were calculated. The analyzed areas covered the trabecular region at 500 µm distal to the growth plate. Each dot represented each animal. Scale bar = 200 μm, *p < 0.05 indicated statistical significance. Trabecular bone (TB), Cortical bone (CB).

**Figure 8:**
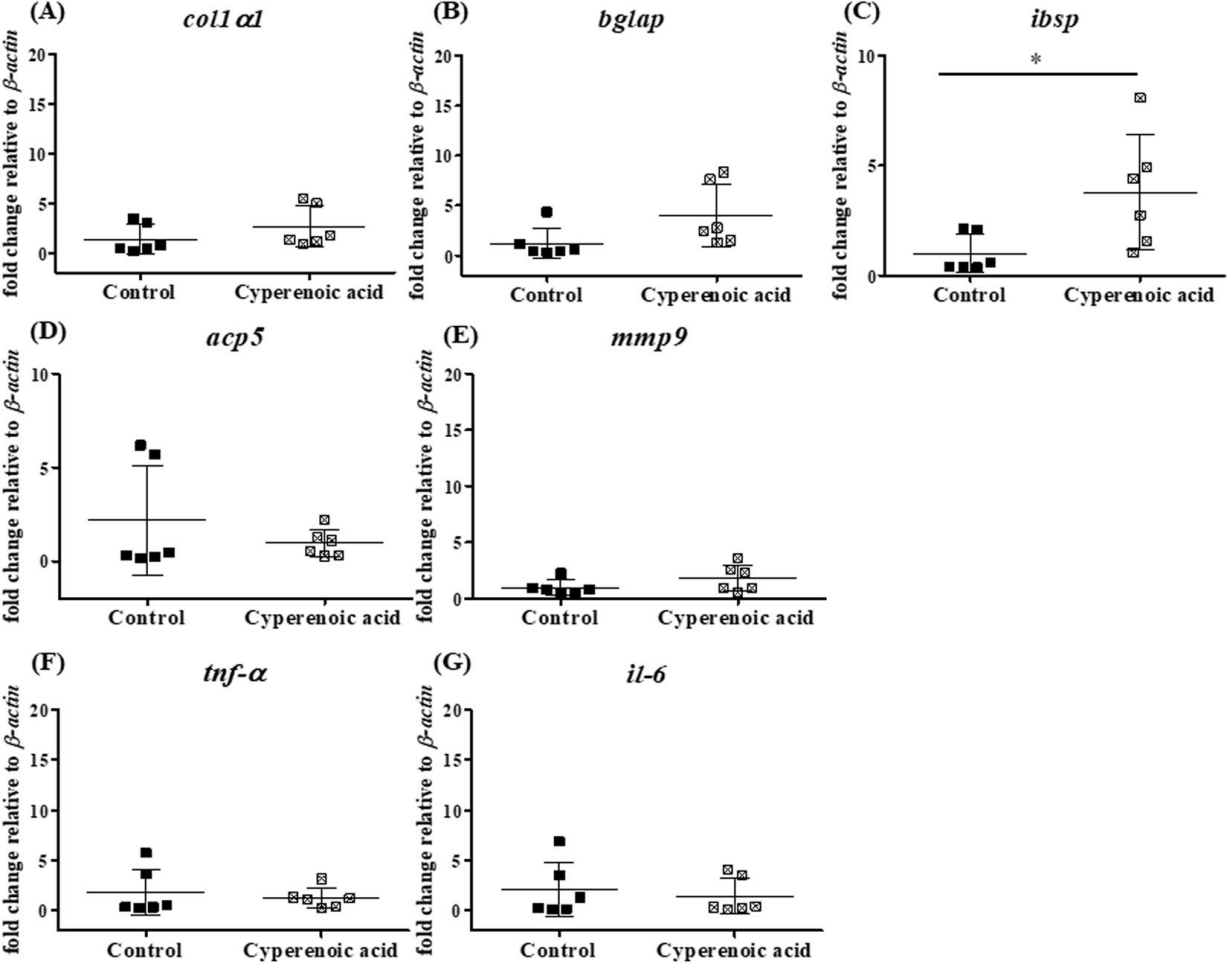
Effect of cyperenoic acid on expression of osteoblast related genes, osteoclast related genes and pro-inflammatory genes in SAMP6 mice. (**A**–**G**) SAMP6 mice were fed with the standard diet food AIN-93M or the standard diet food supplemented with 0.01% cyperenoic acid for 19 weeks. The relative level of mRNA of osteoblast related genes, osteoclast related genes and pro-inflammatory genes in bone marrow cells were detected by RT-qPCR. Each dot represented each animal. *p < 0.05 indicated statistical significance.

To determine the effect of cyperenoic acid on bone loss in SAMP6 mice, femur bones were subjected to bone histomorphometric measurement after 19 weeks of feeding with cyperenoic acid-supplemented diet. As shown in Fig. 7A and B, cyperenoic acid treatment clearly increased the area of trabecular bone, compared to the control group. To quantitatively evaluate the effect of cyperenoic acid on bone loss in SAMP6 mice, bone histomorphometry was performed to obtain four essential trabecular bone parameters. As shown in Fig. 7C–D, the bone volume fraction and trabecular thickness showed an increasing trend in cyperenoic acid-treated group, but the difference did not reach statistical significance. On the other hand, the trabecular separation was significantly less while the trabecular number was significantly increased in the cyperenoic acid-treated group as compared to the control group (Fig. 7E and F), indicating that cyperenoic acid was able to improve bone microstructure. A decrease in trabecular separation was also consistent with the anti-osteoclastic effect of this compound since an increased trabecular separation is often due to the enhanced osteoclast activity. Next, the mRNA expression of genes related to bone formation (*col1a1*, *bglap*, *ibsp*), bone resorption (*acp5*, *mmp9*) and pro-inflammatory cytokines (*tnf-α*, *il-6*) were detected in bone marrow cells isolated from SAMP6 mice (Fig. 8). Cyperenoic acid treatment enhanced the expression of *ibsp*, a gene encoding bone sialoprotein (BSP) which is an osteoblast-related gene and plays an important role in bone formation and bone mineralization (Fig. 8C). On the other hand, this compound did not have any detectable effect on the levels of mRNA of other genes detected here. Taken together, cyperenoic acid supplement in diet before the onset of bone loss in SAMP6 mice significantly delayed bone loss *in vivo*.

## Discussion

*C*. *crassifolius* is a medicinal plant distributed mainly in the southeast Asia, especially in Thailand, Laos, Vietnam and China. The ethanol extract from this plant was demonstrated to have anti-nociceptive and anti-inflammatory effects^31^. Cyperenoic acid belongs to a group of terpenoids found in the extract from *C*. *crassifolius* and has been identified by Boonyarathanakornkit *et al*. (1987)^22^ to possess anti-angiogenesis activity^23,24^. In this study, we reported the novel biological activity of this natural compound against RANKL-induced osteoclast differentiation, its possible mode of action and the effect *in vivo* on senile bone loss.

Cyperenoic acid suppressed RANKL-mediated osteoclast differentiation in BMs with the IC_50_ of 36.69 µM ± 1.02. This compound showed no toxicity against osteoclast precursors, macrophage like cell line and osteoblast precursor in both short term and long term culture (up to 14 days). In addition, upon adding to a mouse diet and continuous feeding to mice for 19 weeks, no apparent organ toxicity was observed. Therefore, this compound shows low toxicity both *in vitro* and *in vivo* at least in mice.

The anti-inflammatory activity of cyperenoic acid was tested in LPS/IFNγ-activated macrophage-like cell line RAW264.7. The signaling downstream of LPS and its receptor shares some pathways with those utilized by RANK/RANKL for osteoclast differentiation^8,32^. Therefore, by preliminarily examining the anti-nitric oxide production in macrophages may help identifying the step(s) affected by cyperenoic acid treatment. In fact, the results obtained from signaling pathway analysis corroborated that cyperenoic acid did not interfere with MAPK and canonical NF-κB pathways.

The molecular mechanism underlying the anti-osteoclastogenic activity of cyperenoic acid appeared to be partly at the non-canonical NF-κB signaling, while other signaling pathways, *i*.*e*. MAPK and canonical NF-κB pathways were largely unaffected by cyperenoic acid treatment. The non-canonical NF-κB signaling pathway is utilized by various cytokines, including RANK/RANKL, LTβ/LTβR and CD40/CD0L^10^. The immediate outcome of the non-canonical NF-κB signaling is the dimerization of partially degraded p52 and RelB and the translocation of this dimer to nuclei which, in turn, regulates target gene expression. TRAF2 and 3 are adaptor proteins that function to regulate the stability of NIK kinase via recruitment of cIAP1/2^33^. Cyperenoic acid treatment decreased the phosphorylation of p100, the appearance of p52 and also transiently affected the level of TRAF3 (Fig. 4). We proposed that cyperenoic acid may affect the activity of enzyme(s) that regulate phosphorylation of p100. The processing of p100 is critical for governing the development of lymphoid organs and bone metabolism^11^. Iotsova *et al*.^12^ found that double knockout mice lacking both p50 and p52 exhibited an osteopetrotic phenotype while single knockout of p50 or p52 did not show bone formation abnormalities. Nevertheless, BMMs cells from *p50*^−/−^*p52*^+/−^ mice differentiate and remain as osteoclast progenitors but the mature osteoclasts could not be detected. Interestingly, p50/p52 double KO mice show severe osteopetrosis and the overexpression of NFATc1 and c-Fos rescues this phenotype, indicating a crucial role of non-canonical NF-κB signaling in RANKL-induced osteoclastogenesis^34,35^. Cyperenoic acid treatment indeed substantially reduced the expression of both proteins in the presence of RANKL. These results strongly indicated that this compound affects the activation of the non-canonical NF-κB signaling and its downstream target genes involved in osteoclast differentiation.

NFATc1 is a master regulator of osteoclast differentiation which control the expression of osteoclast-specific genes encoding key enzymes required for osteoclast functions such as TRAP and cathepsin K via c-Fos^36^. NFATc1-deficient mice exhibit osteopetrotic phenotype because of the defect in osteoclastogenesis process^37^. Some studies reported that in an early stage of osteoclast differentiation, c-Fos is a necessary factor for the early activation of NFATc1 in osteoclastogenesis^38^. Cyperenoic acid treatment completely suppressed the activation of c-Fos at 48 h and this effect was translated to decreased NFATc1 level at 72 and 96 h. Expression of genes under the regulation of NFATc1 and c-Fos were all reduced by cyperenoic acid treatment, including *acp5* and *ctsk*. Interestingly, cyperenoic acid treatment affected not only the positive regulators of osteoclast differentiation but also the negative regulator *irf8*. *irf8* encodes a key modulatory protein for osteoclast differentiation. IRF8 suppresses the function and expression of NFATc1 and its level is reduced during osteoclast differentiation. In mice lacking of *ifr8*, a severe osteoporosis was manifested because of increased in osteoclast numbers^30^. Therefore, cyperenoic acid treatment suppresses the non-canonical NF-κB signaling and affects the expression of the target genes involved in both positive and negative regulation of osteoclast differentiation.

Cell-cell fusion is a critical step to generate MNCs during osteoclast differentiation. Various molecules were identified to play roles during this process such as Dendritic cell–specific transmembrane protein (DC-STAMP). DC-STAMP is under the regulation of NFATc1 and c-Fos^39^. In this study, cyperenoic acid reduced the numbers of TRAP+ cells with 3 or more nuclei during RANKL-induced osteoclast differentiation (Fig. 2D). Because the expressions of NFATc1 and c-Fos were compromised by cyperenoic acid treatment, it is highly likely that the expression of DC-STAMP and the step of cell-cell fusion leading to MNCs are also suppressed.

The effect of cyperenoic acid on functions of osteoclasts was evaluated by bone resorption assay. Addition of this compound at the same time as RANKL stimulation partially reduced the bone resorption areas (Fig. 5). This is an unexpected observation because the differentiation of osteoclasts was almost completely suppressed *in vitro* by TRAP staining with the same concentration of cyperenoic acid. The assay used in this study measured only the areas of resorption but not the depth of the resorbed pits^40^. It is possible that cyperenoic acid treatment may also affect the depth of mineralization. When cyperenoic acid was added after osteoclast differentiation, it also reduced the resorption areas, indicating that this compound may possess bone resorption activity independently of the osteoclast differentiation. How cyperenoic acid influences bone resorption remains unanswered.

Our studies provided compelling evidence that cyperenoic acid may be beneficial for senile osteoporosis in SAMP6 mice. SAMP6 is proposed to be a useful senile osteoporosis mouse model which shows sign of bone loss in relatively young age^41–43^. In our experiment, we supplemented the compound in the regular animal diet and allowed the animals free access to the diet. The delay in bone loss was clearly shown after long term consumption of modified diets with no apparent organ toxicity. Among the four bone microstructural parameters as analyzed by static bone histomorphometry, trabecular separation and trabecular number were significantly improved in cyperenoic acid-treated group. The bone volume and the trabecular thickness increased slightly but did not reach statistical significance between the two groups. These two latter parameters may need longer time of treatment to exhibit the improvement effect; however, an increased trabecular number and a decreased separation clearly confirmed that cyperenoic acid had beneficial effects on bone microstructure. Whether cyperenoic acid treatment can delay bone loss in other strains of mice or in other mouse model of osteoporosis induced by other manipulation needs further investigation.

Interestingly, cyperenoic acid enhanced expression of *ibsp* which encodes the bone sialoprotein (BSP). BSP is synthesized mainly by chondrocytes, a subset of osteoblasts, and osteoclasts. Mice with targeted deletion of *ibsp* show diminished bone growth and mineralization lead to reduce bone formation^44^, indicating that BSP is important for bone mineralization and bone formation. Further, BSP is a useful diagnostic marker of bone resorption reflecting osteoclast activity in human or animal serum^45^. In our study, cyperenoic acid slightly decreased BSP level in SAMP6 mice serum but did not reach the level of statistical significance (data not shown). In a study by Li *et al*.^20^, berberine, an isoquinoline alkaloid, diminished concentration of dexypyridinoline (Dpd) which is a bone resorption marker in urine, but it shows no effect on procollagen type I carboxyterminal extension peptide (PICP) in serum of SAMP6 mice^20^. The effect of cyperenoic acid treatment at the molecular level was examined by detecting gene expression in bone marrow cells in this study. Because our *in vitro* results indicated that cyperenoic acid affects early stage of osteoclast differentiation from BMs, we hypothesized that the cyperenoic acid treatment *in vivo* may affect gene expression profiles of osteoclast precursors reflecting bone formation and resorption. Unexpectedly, we did not find any significant difference in the mRNA level of genes involved in bone resorption. Thus, more careful analysis of gene expression in a well defined cell subsets such as bone marrow stromal cells or osteoblasts and osteocytes may yield more informative results on the effect of this compound on bone formation.

In this study, cyperenoic acid has no impact on ALP activity of pre-osteoblast cell (MC3T3-E1) treated with ascorbic acid. Some natural compounds are shown to affect both osteoclast differentiation and osteoblast maturation/function^46^. Therefore, cyperenoic acid may indirectly affect the expression of *ibsp*. Cyperenoic acid treatment did not have any effect on the expression of *il-6* and *tnf-α* which are related to inflammatory bone loss^47^. Furthermore, histomorphometric parameters are used to distinguish the bone condition. Typically, osteoporosis shows high and low level in bone turnover and bone volume, respectively^29^.

Taken together, we presented evidence that cyperenoic acid from *C*. *crassifolius* interferes with RANKL/RANK signaling at least at the non-canonical NF-kB pathway and the osteoclast differentiation process and might be a potential lead compound for osteoporosis therapeutic and prevention in senile osteoporosis.

## Electronic supplementary material

Supplementary Information
