# Supplementary material for: Cyperenoic acid suppresses osteoclast differentiation and delays bone loss in a senile osteoporosis mouse model by inhibiting non-canonical NF-κB pathway
Source: Sci Rep. 2018 Apr 4;8:5625. doi: 10.1038/s41598-018-23912-3 (PMC5884777; doi:10.1038/s41598-018-23912-3)
Supplement: Supplementary file 1 — Supplementary Information [file 41598_2018_23912_MOESM1_ESM.docx]

Supplementary Information

Cyperenoic acid suppresses osteoclast differentiation and delays bone loss in a senile osteoporosis mouse model by inhibiting non-canonical NF-κB pathway

Supatta Chawalitpong^a^, Ratchanaporn Chokchaisiri^b^, Apichart Suksamrarn^c^, Shigeru Katayama^d^, Takakazu Mitani^e^, Soichiro Nakamura^d^, Ahmad AI Athamneh^d^, Patcharee Ritprajak^f^, Asada Leelahavanichkul^g^, Ratchaneevan Aeimlapa^h,i^, Narattaphol Charoenphandhu ^h,i,j^, Tanapat Palaga^k,l^ *

Table 1 List of primers used in this study

| Gene | Primer sequence (from 5’ to 3’) | | Annealing temp. | Product size |
| --- | --- | --- | --- | --- |
|  | Forward | Reverse | (°C) | (bp) |
| *irf8* | GGAAAGCCTT  ACCTGCTGAC | AAGGTCACC  GTGGTCCTT | 55 | 112 |
| *ctsk* | GGCCAACTC  AAGAAGAAA | GTACCCTCT  GCATTTAGC | 58 | 225 |
| *nfatc1* | GGTAACTCTGTCTT  TCTAACCTTAAGCTC | GTGATGACCCCAGCA  TGCACCAGTCACAG | 62 | 240 |
| *cfos* | CTCCTTCTCC  AGCATGGGC | ACCACAGTC  CATGCCATC | 60 | 81 |
| *actb*  (1) | ACCAACTGGGAC  GACATGGAGAA | GTGGTGGTGA  AGCTGTAGCC | 55 | 380 |
| *gapdh* | TCCACCACC  CTGTTGCTG | ACCACAGTC  CATGCCATC | 60 | 452 |
| *il-6* | AGTCCGGAGA  GGAGACTTCA | ATTTCCACGA  TTTCCCAGAG | 58 | 108 |
| *tnf-α* | GGCAGGTCTACT  TTGGAGTCATTGC | ACATTCGAGGCT  CCAGTGAATTCGG | 60 | 300 |
| *col1α1* | CTTGGTGGTTTT  GTATTCGATGAC | GCGAAGGCA  ACAGTCGCT | 55 | 101 |
| *lbsp* | GCACTCCAAC  TGCCCAAGA | TTTTGGAGCC  CTGCTTTCTG | 60 | 51 |
| *bglap* | CTGACAAAGCC  TTCATGTCCAA | GCGGGCGAGT  CTGTTCACTA | 50 | 59 |
| *mmp9* | GTCTTCCTGG  GCAAGCAGTA | CTGGACAGAA  ACCCCACTTC | 58 | 115 |
| *acp5* | CCAATGCCAA  AGAGATCGCC | TCTGTGCAGAG  ACGTTGCCAAG | 56 | 216 |
| *actb*  (2) | CACTATTGGCA  ACGAGCGGTTC | ACTTGCGGTG  CACGATGGAG | 55 | 249 |

**
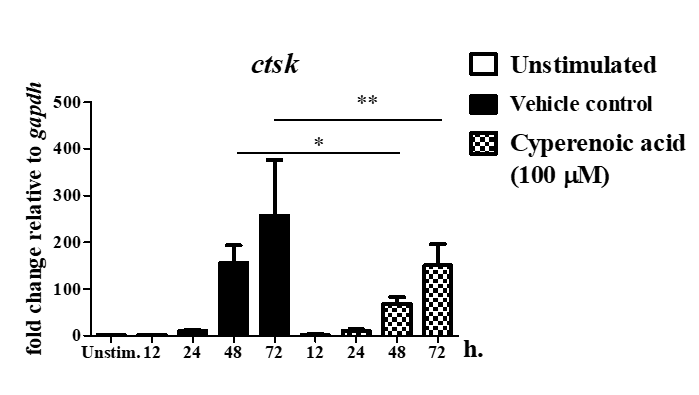
**

**Supplementary Figure 1**. Effect of cyperenoic acid on *ctsk* expression

BMs were treated as described for Figure 3A-E. Total RNA was extracted and subjected to RT-qPCR and normalized by *gapdh*. The data are representative of three independent experiments and presented as the mean ± S.D. ** *p*<0.01 and * *p*< 0.05 indicated statistical significance.

**
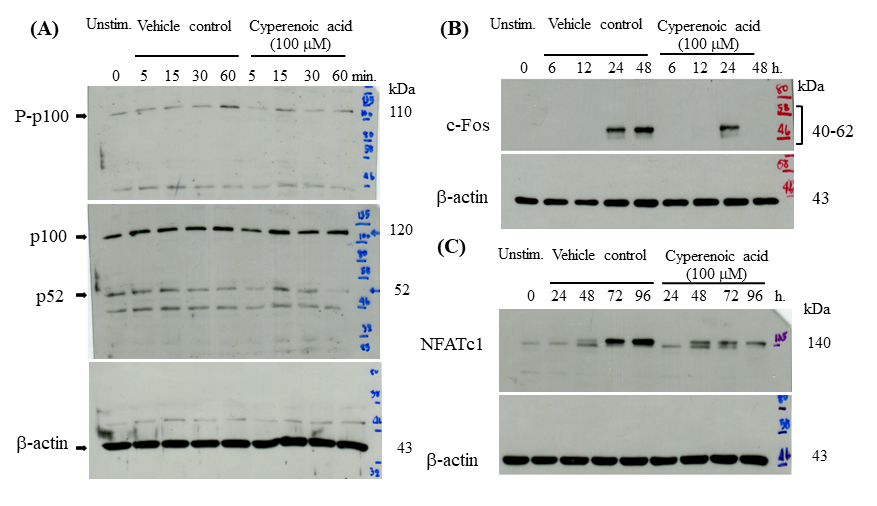
**

**Supplementary Figure 2**. Effect of cyperenoic acid on activation of p100/52 and expression of c-Fos and NFATc1 by Western blot

BMs were treated as described for Figure 4B and 4E. Whole cell lysates were analyzed for p100/52 (A), c-Fos (B) and NFATc1 (C) by Western blot. β-actin was used as a loading control. Data were representative of three independent experiments.

**
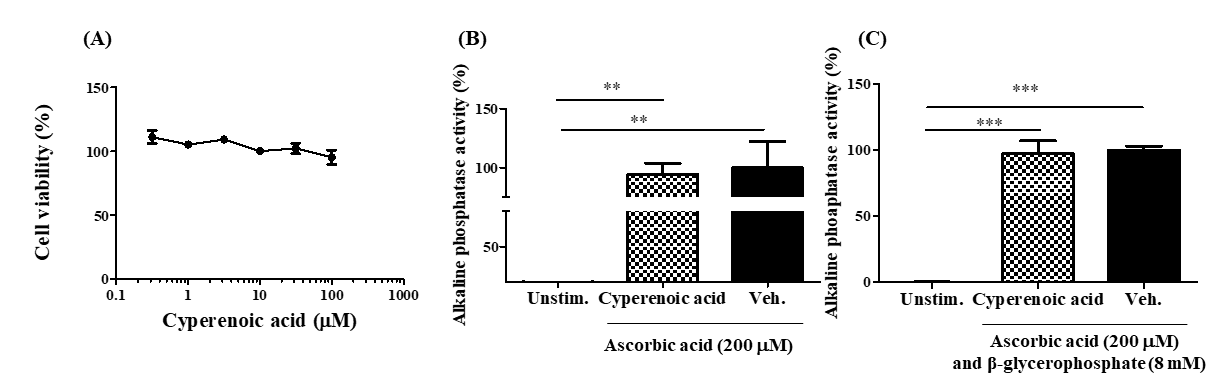
**

**Supplementary Figure 3.** Effect of cyperenoic acid on viability of pre-osteoblast and osteoblast differentiation

(A) Toxicity of cyperenoic acid on osteoblast precursor cell line MC3T3-E1 was examined by treating cells with various concentrations of cyperenoic acid (0-100 μM) for 24 h and cell viability was measured by MTT assay. The IC_50_ of cyperenoic acid was ≥ 100 μM. (B) MC3T3-E1 cells were pre-treated with cyperenoic acid (100 μM) or vehicle control DMSO and stimulated with ascorbic acid (200 μM) or (C) ascorbic acid (200 μM) and β-glycerophosphate (8 mM) for 10 days. The alkaline phosphatase activity was measured and normalized by the protein concentration in each sample. The data are representative of three independent experiments and presented as the mean ± S.D. ***p* <0.01 and ***p* <0.001 indicated statistical significance.


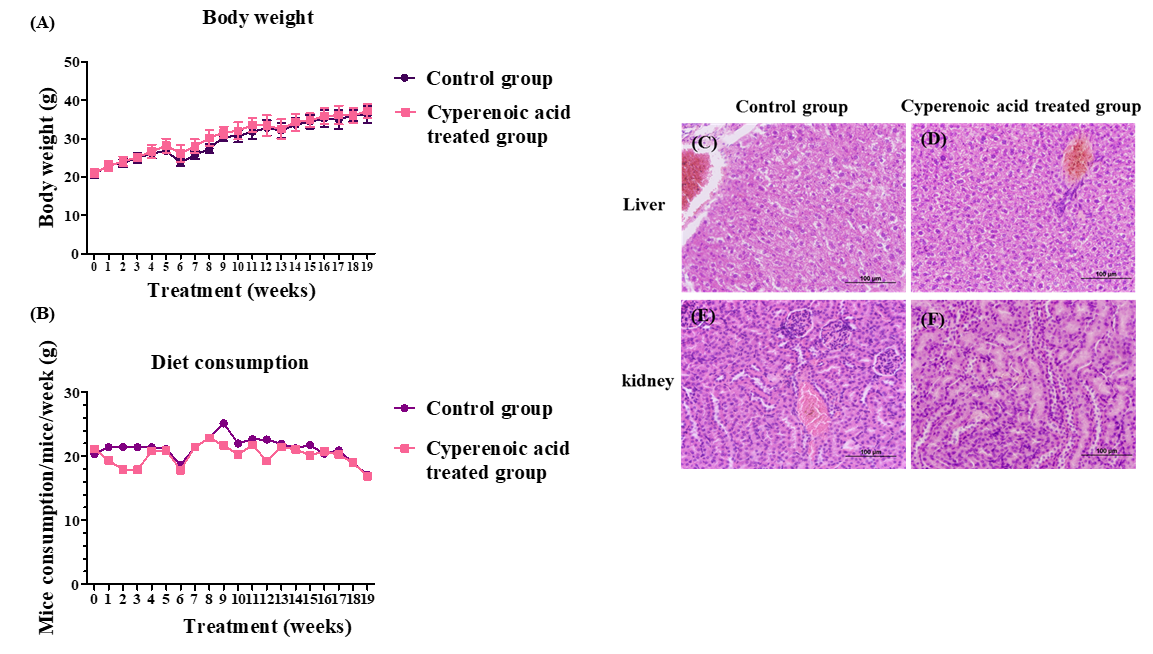


**Supplementary Figure 4.** Effect of cyperenoic acid containing diet on body weight, diet consumption and histomorphology of liver and kidney

Changes in body weight (A) and diet consumption (B) in SAMP6 mice fed with normal chow containing 0.01% cyperenoic acid or absolute ethanol for 19 weeks. (C-F) Histological analysis with H&E staining, (C-D) either liver sections (E-F) or kidney sections were used to indicate side effect. Scale bar = 100 μm.
